# Further evidence for population specific differences in the effect of DNA markers and gender on eye colour prediction in forensics

**DOI:** 10.1007/s00414-016-1388-2

**Published:** 2016-05-24

**Authors:** Ewelina Pośpiech, Joanna Karłowska-Pik, Bartosz Ziemkiewicz, Magdalena Kukla, Małgorzata Skowron, Anna Wojas-Pelc, Wojciech Branicki

**Affiliations:** 1 Institute of Zoology, Faculty of Biology and Earth Sciences, Jagiellonian University, Kraków, Poland; 2 Malopolska Centre of Biotechnology, Jagiellonian University, Kraków, Poland; 3 Faculty of Mathematics and Computer Science, Nicolaus Copernicus University, Toruń, Poland; 4 Faculty of Biochemistry, Biophysics and Biotechnology, Jagiellonian University, Kraków, Poland; 5 Department of Dermatology, Collegium Medicum of the Jagiellonian University, Kraków, Poland

**Keywords:** Forensic DNA Phenotyping (FDP), Eye colour prediction, Gender effect, IrisPlex SNPs, Neural networks, Logistic regression

## Abstract

The genetics of eye colour has been extensively studied over the past few years, and the identified polymorphisms have been applied with marked success in the field of Forensic DNA Phenotyping. A picture that arises from evaluation of the currently available eye colour prediction markers shows that only the analysis of *HERC2-OCA2* complex has similar effectiveness in different populations, while the predictive potential of other loci may vary significantly. Moreover, the role of gender in the explanation of human eye colour variation should not be neglected in some populations. In the present study, we re-investigated the data for 1020 Polish individuals and using neural networks and logistic regression methods explored predictive capacity of IrisPlex SNPs and gender in this population sample. In general, neural networks provided higher prediction accuracy comparing to logistic regression (AUC increase by 0.02–0.06). Four out of six IrisPlex SNPs were associated with eye colour in the studied population. *HERC2* rs12913832, *OCA2* rs1800407 and *SLC24A4* rs12896399 were found to be the most important eye colour predictors (*p* < 0.007) while the effect of rs16891982 in *SLC45A2* was less significant. Gender was found to be significantly associated with eye colour with males having ~1.5 higher odds for blue eye colour comparing to females (*p* = 0.002) and was ranked as the third most important factor in blue/non-blue eye colour determination. However, the implementation of gender into the developed prediction models had marginal and ambiguous impact on the overall accuracy of prediction confirming that the effect of gender on eye colour in this population is small. Our study indicated the advantage of neural networks in prediction modeling in forensics and provided additional evidence for population specific differences in the predictive importance of the IrisPlex SNPs and gender.

## Introduction

Genetic prediction of human traits known as Forensic DNA Phenotyping (FDP) has enabled further development of methods and tools offered for intelligence purposes. Detailed description of appearance of an unknown individual based on DNA analysis of biological material can streamline the process of investigation in criminal cases without any suspects or entries in DNA profile databases [1–3]. Eye colour has been one of the first traits of human appearance successfully applied in the field of FDP. Among several available eye-colour prediction tools, the IrisPlex model is based on the largest number of samples (>9000 Europeans) [4] and was successfully validated by a multicenter EDNAP study [5]. IrisPlex involves examination of six DNA variants, namely rs12913832 in *HERC2*, rs1800407 in *OCA2*, rs12896399 in *SLC24A4*, rs16891982 in *SLC45A2*, rs1393350 in *TYR* and rs12203592 in *IRF4* and has been introduced to predict blue and brown eye colour precisely [6]. There is significant evidence from functional studies that the position rs12913832 in *HERC2* plays a crucial role in blue eye colour determination [7], and this position has also been confirmed as the key eye colour predictor in various population samples. Predictive capacity of the remaining IrisPlex SNPs is not so obvious and shows significant variation depending on a study population [8–12]. Pietroni et al., for instance, argued that three SNPs: rs12913832, rs1800407 and rs16891982 are the only informative markers among the six IrisPlex polymorphisms [13]. Position rs12203592 is regarded as the weakest predictor, and its association with eye colour has not been confirmed in several study samples [8, 10, 12, 14]. In our population sample of 718 individuals from Poland, only four IrisPlex SNPs have been found to be associated with eye colour, with *IRF4* and *TYR* unimportant for prediction [11].

Moreover, the examination of a Spanish population sample has unexpectedly detected the significance of gender in the explanation of human eye colour variation [9]. Gender has been found to explain discrepancies in eye colour prediction based on *HERC2* rs12913832 polymorphism indicating that females tend to have darker eye colour than males when comparing the same genotypes. The intriguing observation based on the Spanish samples has been later confirmed in Italian population where gender has been ranked as the second most important predictor [13]. However, this effect has not been confirmed in samples from Denmark and Sweden [13]. Moreover, gender has been found not to improve the prediction of eye colour when incorporated into the original IrisPlex model [15] suggesting that the effect of gender on eye colour may be population specific and stronger in populations of southern Europe.

In the present study, the role of the six IrisPlex SNPs and gender for eye colour prediction was investigated in the population of 1020 previously genotyped individuals from Poland using neural networks (NN) and logistic regression modeling. Additionally, the IrisPlex SNPs were analysed with the available online IrisPlex calculator in order to compare its predictive performance in males and females.

## Materials and methods

### Population samples and genotyping

Samples involved 1020 unrelated individuals (>18 years old) from Poland previously genotyped and interpreted using different statistical methods [11, 16]. The study cohort included 420 males (41.2 %) and 597 females (58.5 %). No data about gender was available for the remaining three samples (0.3 %). Eye colour of the participants has been assessed by a physician specializing in dermatology and categorized as blue, green, hazel and brown. Written informed consent was obtained from all the samples donors, and the study protocol was approved by the Ethics Committee of the Jagiellonian University in Krakow (KBET/17/B/2005) and the Commission on Bioethics of the Regional Board of Medical Doctors in Krakow (48 KBL/OIL/2008). The genotyping procedure for the total number of 24 SNPs in 11 genes has been described in Pośpiech et al. [16].

### Evaluation of the effect of SNPs and gender on eye colour in the study population

The genotypes for the six IrisPlex SNPs (rs12913832, rs1800407, rs12896399, rs16891982, rs1393350, rs12203592), gender and eye colours were retrieved and subjected to association testing which involved the pooled training and testing set from the previous study [11] (the total number of 1020 individuals) and prediction modeling using neural networks and logistic regression approaches. Analyses were performed with IBM SPSS Statistics v. 23 (SPSS Inc., Chicago, IL, USA). The bundle includes IBM SPSS Regression and IBM SPSS Neural Networks modules.

#### Dependence testing

The dependencies between eye colour (defined as blue/green/hazel/brown) and gender were tested using Pearson’s *χ* ^2^ test. Pearson’s contingency coefficient (more precisely its adjusted version with maximal value equal to 1) and Cramér’s V coefficient were calculated to evaluate the strength of association of eye colour and all independent variables (6 IrisPlex SNPs and gender), all of them treated as categorical attributes [17].

#### Power of the sample size

Minimal values of odds ratios (ORs) detectable with a power of at least 80 % were assessed with Fisher’s exact test using power and sample size program (PS Program) v.3.1.2 (http://biostat.mc.vanderbilt.edu/wiki/Main/PowerSampleSize).

#### Association testing

Binary logistic regression was applied to evaluate the association of particular variables with eye colour defined as blue/non-blue. Analyses were performed on the entire set of 1020 samples. Variables were tested as single factors (univariate logistic regression analysis) and then analysed simultaneously (multivariate logistic regression analysis) to assess the influence of gender on the six IrisPlex SNPs effects. Allelic ORs with 95 % confidence intervals (CIs) and respective *p* values were estimated for minor alleles categorized in an additive manner. The seven tested variables were ranked according to their importance using −2log likelihood of reduced model statistic. The proportion of total variance in eye colour explained by the tested variables was estimated using Nagelkerke pseudo-*R* ^*2*^ statistic. Statistical significance was set at *p* value lower than 0.05. However, the results of association testing considering Bonferroni correction for multiple comparisons were also discussed (*p* < 0.007).

#### Prediction modeling

The entire set of 1020 individuals was used to develop prediction models and test the effect of particular SNPs and gender on prediction. A few prediction methods, including logistic regression, neural networks, classification and regression trees and random forests, were used to build models that were tested using tenfold cross-validation procedure. For this purpose, the data was split randomly into 10 equal-sized parts (identically for all tested models). For each *k* (*k = 1,2,…,10*) the *k*th part was excluded, and the model was built using the data from the others *k − 1* parts. Then the prediction error was calculated on the excluded *k*th part of the data. The final prediction error was estimated by the mean of errors of 10 models built in the cross-validation procedure. Because its lowest values were achieved for neural networks and well known logistic regression procedure, we decided to concentrate only on these two prediction methods.

By the neural network, we mean multilayer perceptron (MLP) with one hidden layer and an automatically selected number of neurons. The activation functions were hyperbolic tangent for the hidden layer and softmax for the output layer. Synaptic weights were updated after passing all training data (batch training type). As an optimization algorithm, we used scaled conjugate gradient with default initial parameters of IBM SPSS Statistics. The multinomial logistic regression model was developed using block entry of variables (entry value 0.05). The utility of the particular IrisPlex SNPs and gender for eye colour prediction in both cases was assessed by the sequential implementation of variables into the prediction models and the calculation of prediction accuracy parameters including area under the ROC curve (AUC), sensitivity and specificity [e.g. [18, 19]. All the described analyses were performed with IBM SPSS Statistics v. 23.

#### Prediction testing with online IrisPlex calculator

All the collected samples were tested with online web-based IrisPlex model (http://hirisplex.erasmusmc.nl/). Probabilities for particular eye colours were generated for the tested 1020 samples considering all six SNPs and position rs12913832 alone (assuming unknown state for the remaining 5 SNPs). Sensitivity and specificity of prediction were calculated separately for males and females, and the dependence of their values and gender was tested using Pearson’s *χ* ^2^ test. Additionally, AUC values for particular eye colours were calculated to compare performance of IrisPlex model and prediction models developed under this study. Due to the fact that IrisPlex tool considers only three eye colour categories, hazel eye colour category was combined in this procedure with brown eye colour category because of their highest similarity [19].

## Results

### Study sample characteristics

**Fig. 1:**
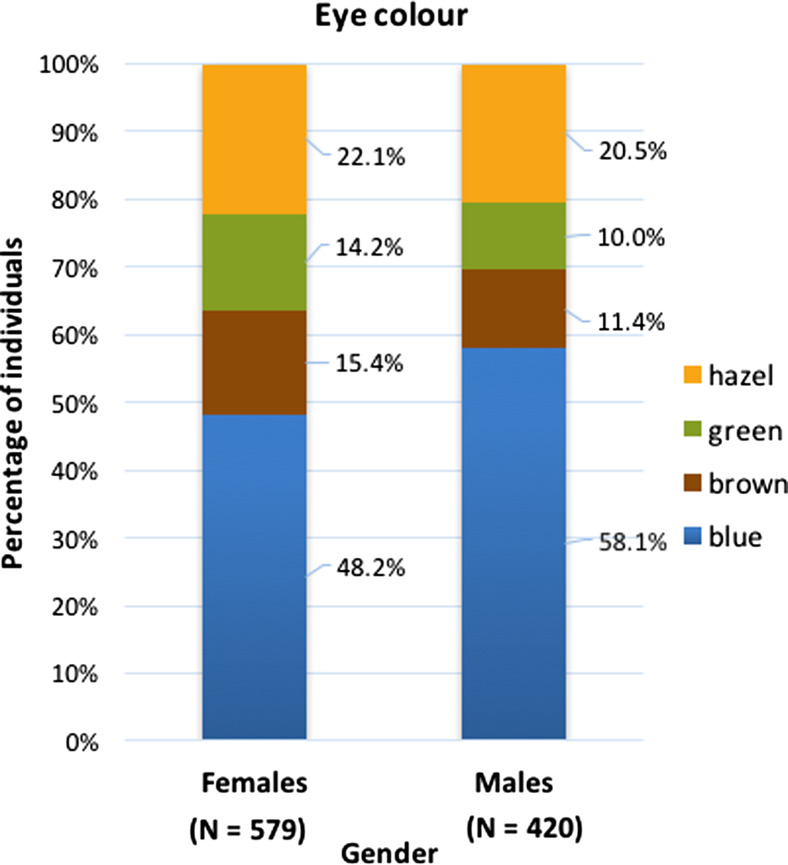
Eye colour frequencies in females and males in the Polish study sample

The Polish population under study comprised of 535 (52.5 %) individuals of blue eye colour, 127 (12.5 %) individuals of green eye colour, 218 (21.4 %) individuals of hazel eye colour and 140 (13.7 %) individuals of brown eye colour. The prevalence of blue eye colour was found to be significantly higher in males (58.1 %) than in females (48.2 %) with *χ* ^2^*p* = 0.002. In contrast, females were found more likely to have green eye colour (14.2 %) comparing to males (10.0 %) with *χ* ^2^*p* = 0.044. No significant differences were noted between genders for hazel and brown eye colours (*χ* ^2^*p* = 0.532 and *χ* ^2^*p* = 0.070, respectively) (Fig. 1).

### Correlation testing between gender, SNPs and eye colour

Correlation analysis between particular variables and eye colour showed the strongest effect (correlation coefficient >0.5) for rs12913832 in *HERC2* on blue/green/hazel/brown eye colour as well as blue/non-blue eye colour with *p* = 5.711 × 10^−138^ and *p* = 1.189 × 10^−108^, respectively. Small effects (correlation coefficient 0.05–0.2) were noted for rs1800407 in *OCA2*, rs12896399 in *SLC24A4* and rs16891982 in *SLC45A2* with *p* = 5.508 × 10^−4^, *p* = 0.024, *p* = 0.014, respectively, when four eye colour categories were considered and *p* = 1.539 × 10^−4^, *p* = 0.002 and *p* = 0.026, respectively, when blue/non-blue eye colour was studied. No effect of rs1393350 and rs12203592 was noted for both eye colour categorizations. Gender was found to have small size effect on eye colour (correlation coefficient ~0.1) with *p* = 0.010 for blue/green/hazel/brown and *p* = 0.002 for blue/non-blue eye colour categorization (Table 1).

**Table 1:** Correlation analysis between 6 IrisPlex SNPs, gender and eye colour

Variable	Gene	*N*	Correlation analysis					
			Blue/green/hazel/brown eye colour			Blue/non-blue eye colour		
			Correlation coefficient^a^	Effect size^b^	Pearson *p* value	Correlation coefficient^a^	Effect size^b^	Pearson *p* value
rs12913832	*HERC2*	1020	0.566/0.766	Large	5.711 × 10^−138^	0.698/0.810	Large	1.189 × 10^−108^
rs1800407	*OCA2*	1020	0.108/0.185	Small	5.508 × 10^−4^	0.131/0.184	Small	1.539 × 10^−4^
rs12896399	*SLC24A4*	1020	0.084/0.145	Small	0.024	0.112/0.157	Small	0.002
rs16891982	*SLC45A2*	1020	0.089/0.152	Small	0.014	0.085/0.120	Small	0.026
rs1393350	*TYR*	1020	0.045/0.079	None	0.649	0.044/0.063	None	0.368
rs12203592	*IRF4*	1020	0.036/0.061	None	0.861	0.023/0.032	None	0.770
Gender	*–*	1017	0.105/0.148	Small	0.010	0.097/0.137	Small	0.002

^a^Correlation coefficients: Cramér’s V coefficient/adjusted contingency coefficient

^b^Effect size: none—*k x* Cramér’s V coefficient <0.1, small—*k x* Cramér’s V coefficient 0.1–0.3, medium—*k x* Cramér’s V coefficient 0.3–0.5, large—*k x* Cramér’s V coefficient >0.5, where *k x* Cramér’s V coefficient is so called Cohen’s coefficient and *k* is equal to the square root of 2 for blue/green/hazel/brown eye colour and all genes and 1 in other cases [17]

### Power of the sample size

Theoretical minimal ORs values detectable with a power of at least 80 % in a group of 535 blue and 485 non-blue individuals were calculated to equal OR = 1.510 (or 0.627) for rs12913832, OR = 1.925 (or 0.388) for rs1800407, OR = 1.433 (or 0.692) for rs12896399, OR = 2.549 (or 0.132) for rs16891982, OR = 1.509 (or 0.627) for rs1393350 and OR = 1.784 (0.461) for rs12203592 depending on the minor allele frequency.

### Association analyses with logistic regression

Logistic regression was used to test the effect size of association between the tested variables and blue/non-blue eye colour. Firstly, independent effects of seven individual variables were examined in univariate association analyses. Position rs12913832 in *HERC2* was confirmed to be the most strongly associated with eye colour with C allele increasing the odds for blue eye colour by a factor of 32.3 (*p* = 1.961 × 10^−72^). Among six IrisPlex SNPs, significant association was also noted for rs1800407 in *OCA2* (*p* = 4.886 × 10^−5^), rs12896399 in *SLC24A4* (*p* = 5.260 × 10^−4^) and rs16891982 in *SLC45A2* (*p* = 0.009) with *OCA2* and *SLC24A4* polymorphisms significant also after Bonferroni correction. No association was noted for the remaining two polymorphisms, rs1393350 in *TYR* (*p* = 0.158) and rs12203592 in *IRF4* (*p* = 0.675). Gender was found to be significantly associated with eye colour with males having ~1.5 higher odds for blue eye colour comparing to females (*p* = 0.002). Nagelkerke *R* ^*2*^ for gender was 1.3 % (for blue/non-blue eye colour) which is much lower than established for rs12913832 (*R* ^*2*^ = 55.6 %), lower than calculated for rs1800407 (*R* ^*2*^ = 2.3 %) and rs12896399 (*R* ^*2*^ = 1.6 %) but higher than established for rs16891982 (*R* ^*2*^ = 0.9 %), rs1393350 (*R* ^*2*^ = 0.3 %) and rs12203592 (*R* ^*2*^ = 0.02 %). In the next step, all seven variables were tested simultaneously in multivariate association analysis. In this approach, position rs1393350 in *TYR* was also found to be associated with blue/non-blue eye colour (*p* = 0.029). However, this result was not significant after Bonferroni correction for multiple comparisons. The significance of gender in multivariate association analysis increased to *p* = 1.480 × 10^−4^ placing gender as the third most significant factor in blue/non-blue eye colour determination after rs12913832 (*p* = 9.055 × 10^−64^) and rs12896399 (*p* = 7.402 × 10^−7^). Variables were additionally ranked using −2log likelihood of reduced model statistic confirming gender being the third most important factor among the seven tested in blue/non-blue eye colour determination. Gender was ranked as the fourth most important factor (after rs12913832, rs1800407 and rs12896399) when eye colour was categorized as blue/green/hazel/brown (Table 2).

**Table 2:** Logistic regression association testing between six IrisPlex SNPs, gender and eye colour

Variable	Gene	MAF	Univariate association analysis			Multivariate association analysis		Rank for blue/non-blue eye colour		Rank for blue/green/hazel/brown eye colour	
			OR (95 % CI)^a^	*p* value	*R* ^2^ (%)	OR (95 % CI)^a^	*p* value	−2log likelihood of reduced model	Rank	−2log likelihood of reduced model	Rank
rs12913832	*HERC2*	T 0.222	*0.031 (0.021–0.045***)**	*1.961 × 10* ^*−72*^	55.6	*0.020 (0.013–0.032)*	*9.055 × 10* ^*−64*^	798.429	1	1415.422	1
rs1800407	*OCA2*	A 0.064	*0.459 (0.315–0.668)*	*4.886 × 10* ^*−5*^	2.3	*2.430 (1.334–4.427)*	*3.720 × 10* ^*−3*^	252.728	4	684.362	2
rs12896399	*SLC24A4*	A 0.438	*1.373 (1.148–1.642)*	*5.260 × 10* ^*−4*^	1.6	*1.960 (1.502–2.558)*	*7.402 × 10* ^*−7*^	270.239	2	679.576	3
rs16891982	*SLC45A2*	C 0.027	*0.471 (0.268–0.827)*	*0.009*	0.9	*0.379 (0.186–0.772)*	*0.007*	251.326	5	656.226	5
rs1393350	*TYR*	A 0.223	1.162 (0.943–1.431)	0.158	0.3	*1.406 (1.035–1.910)*	*0.029*	249.108	6	656.059	6
rs12203592	*IRF4*	T 0.087	1.069 (0.783–1.460)	0.675	0.02	1.165 (0.752–1.806)	0.494	244.742	7	646.489	7
Gender	–	–	*1.487 (1.156–1.913)*	*0.002*	1.3	*2.042 (1.412–2.953)*	*1.480 × 10* ^*−4*^	259.197	3	662.316	4

^a^OR values have been calculated for blue/non-blue eye colour categorization

Significant results (*p* < 0.05) are marked with italics

### Prediction modeling using neural networks and the complete set of 1020 individuals

**Fig. 2:**
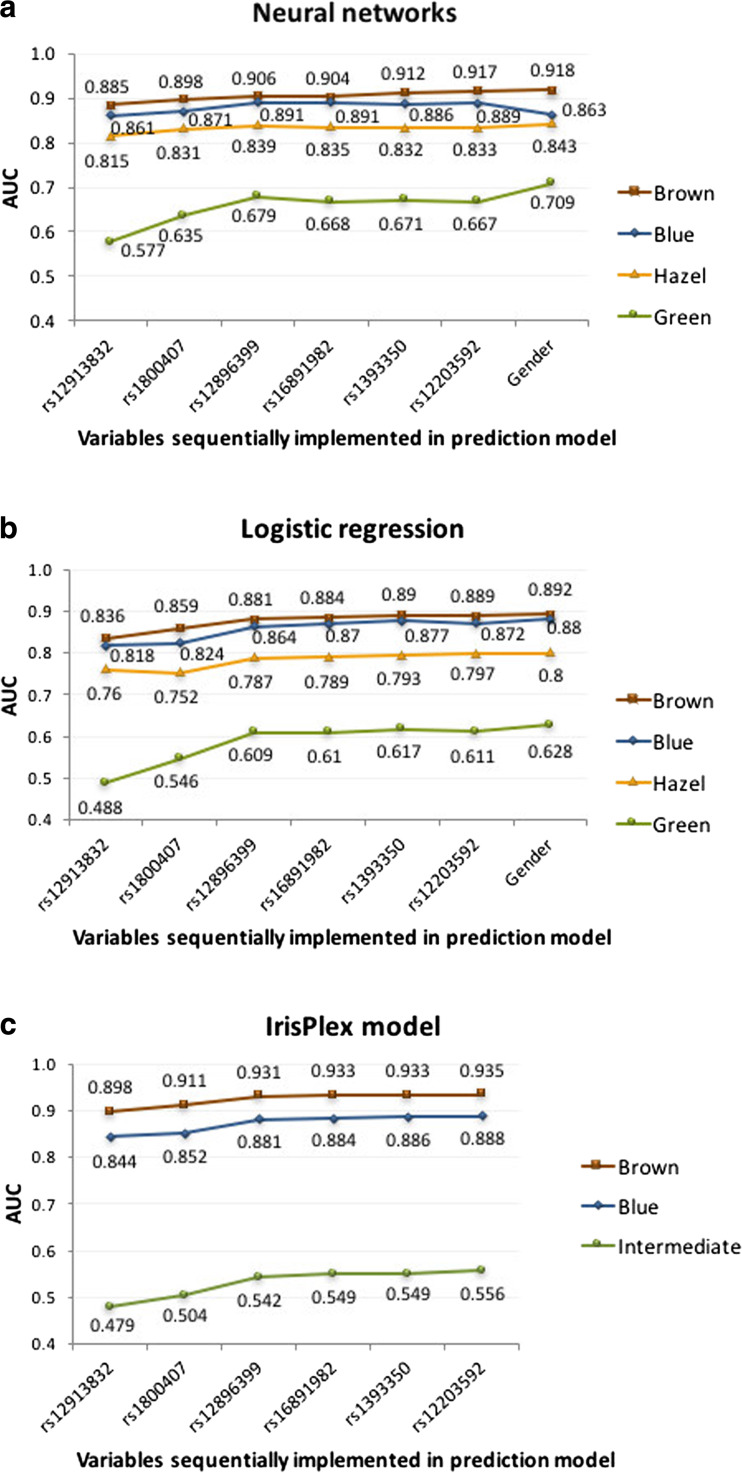
Contribution of six IrisPlex SNPs and gender into the overall accuracy of eye colour prediction measured by AUC. Values of AUC have been calculated for neural networks (**a**), multinomial logistic regression (**b**) and IrisPlex model (**c**)

The entire population of 1020 Polish samples was used to develop neural networks prediction model and evaluate the predictive capacity of IrisPlex SNPs and gender. Variables were sequentially incorporated into the analyses and tested for their impact on accuracy prediction expressed by the value of AUC. As presented in Fig. 2, position rs12913832 in *HERC2* alone provided accurate prediction of blue, brown and hazel eye colour at the level of AUC >0.8. Noticeable impact on AUC increase (>0.01) was observed with rs1800407 in *OCA2* and rs12896399 in *SLC24A4*. Position rs1800407 in *OCA2* increased the value of AUC for green eye colour (increase by 0.058 for neural networks) and brown eye colour prediction (increase by 0.013 for neural networks). Position rs12896399 in *SLC24A4* improved the prediction of blue and green eye colour by increasing the value of AUC by 0.02 and 0.04, respectively. The remaining three polymorphisms had smaller impact on the accuracy of prediction increasing the value of AUC by less than 0.01 (Fig. 2). The implementation of gender into the neural networks prediction model only marginally impacted the accuracy of the prediction measured by AUC, sensitivity and specificity, and the results were ambiguous. Gender noticeably increased the accuracy of green eye colour prediction with AUC change by 0.04 but with the sensitivity decrease from 0.71 to 0.00 %. Similar result was obtained for hazel eye colour (AUC increase by 0.01 and sensitivity decrease by 1.28 percentage points (pp)). Slight decrease in AUC (by 0.026), sensitivity (by 1.55 pp) and specificity (by 0.49 pp) values was noted for blue eye colour when gender was considered. In turn, increase in AUC (by 0.001) and prediction sensitivity (by 0.81 pp) values was noted for brown eye colour category (Fig. 2, Table 3). The small impact of gender on eye colour prediction was also noted when multinomial logistic regression was used with the increase in AUC value for all eye colour categories (change from 0.872 to 0.880 for blue, 0.611 to 0.628 for intermediate, 0.797 to 0.800 for hazel and 0.889 to 0.892 for brown eye colour) but without changes in sensitivity and specificity prediction parameters values (Table 3). Importantly, neural networks method in general provided higher values of AUC comparing to logistic regression with AUC increase from 0.872 to 0.889 for blue eye colour, from 0.611 to 0.667 for green eye colour, from 0.797 to 0.833 for hazel eye colour and from 0.889 to 0.917 for brown eye colour category (Fig. 2, Table 3).

**Table 3:** Impact of gender on the performance of eye colour prediction models developed with a Polish sample set using neural networks and multinomial logistic regression methods

Eye colour category	Prediction accuracy	Mathematical method			
		Neural networks		Multinomial logistic regression	
		Gender not included	Gender included	Gender not included	Gender included
Blue eye colour	AUC	0.889	0.863	0.872	0.880
	Sensitivity [%]	94.06	92.51	93.11	93.11
	Specificity [%]	74.34	73.85	74.07	74.07
Green eye colour	AUC	0.667	0.709	0.611	0.628
	Sensitivity [%]	0.71	0.00	0.00	0.00
	Specificity [%]	99.55	99.88	99.88	99.88
Hazel eye colour	AUC	0.833	0.843	0.797	0.800
	Sensitivity [%]	64.88	63.60	65.20	65.20
	Specificity [%]	81.04	79.91	80.22	80.22
Brown eye colour	AUC	0.917	0.918	0.889	0.892
	Sensitivity [%]	34.23	35.04	33.80	33.80
	Specificity [%]	95.54	94.18	94.52	94.52

### Prediction testing using IrisPlex online tool

**Fig. 3:**
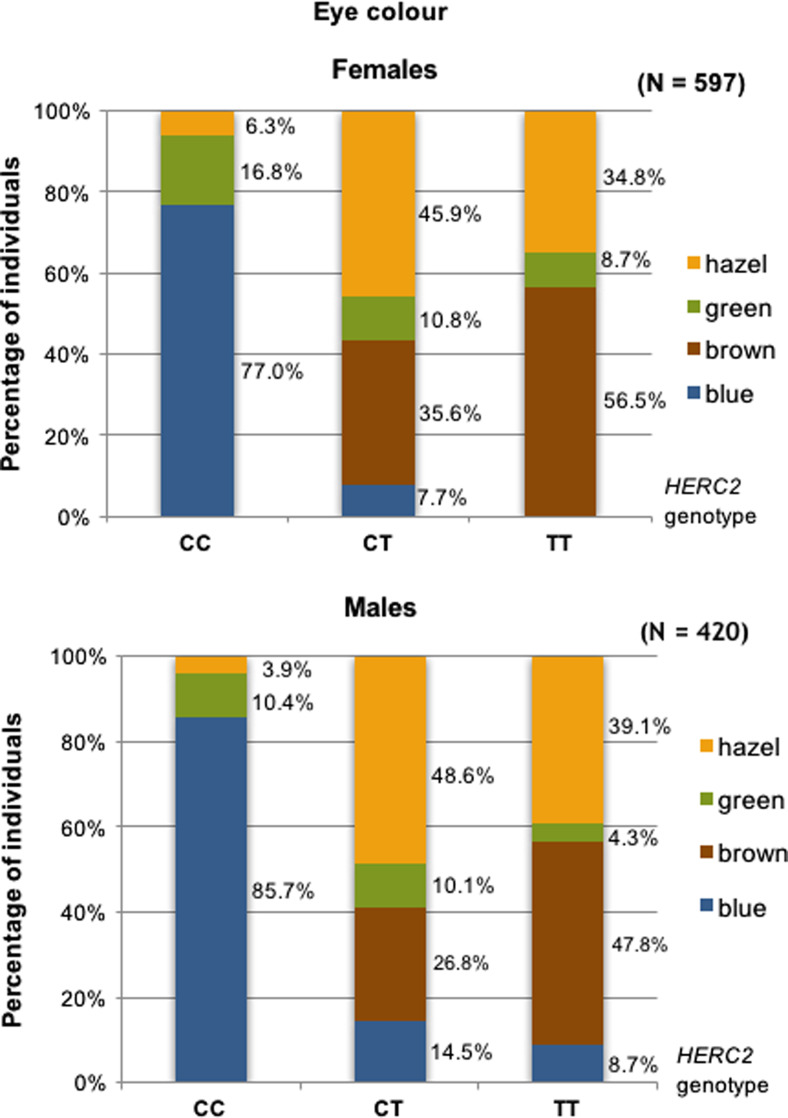
Distribution of eye colour categories in individuals of CC, CT and TT genotype in rs12913832 *HERC2* compared in males and females

Gender has been suggested to explain discrepancies in eye colour prediction based on rs12913832 position in *HERC2* [9]. Therefore, we analysed the distribution of genotypes in males and females and found that among males of CC genotype 85.7 % have blue eye colour but significantly lower proportion of blue eye colour (77 %) has been found in females of CC genotype (*p* = 0.007) (Fig. 3). To further evaluate the effect of gender on eye colour prediction, we tested all 1020 samples with available online IrisPlex model. Higher level of prediction sensitivity of blue eye colour was observed in females (95.1 %) comparing to males (91.8 %) when using genotype data for all six SNPs, and similar result was obtained when the prediction was performed based on rs12913832 position alone (94.1 % of sensitivity prediction in females, 91.0 % of sensitivity prediction in males). However, in both calculations, the results were insignificant (*p* = 0.117 and *p* = 0.170, respectively). Moreover, higher level of specificity of blue eye colour prediction was shown in males (76.1 or 79 % when using six IrisPlex SNPs or rs12913832) comparing to females (71.8 or 73.8 % when using six IrisPlex SNPs or rs12913832), but the results were also insignificant. Overall, no significant differences in eye colour prediction success were observed between genders across all eye colour categories (Tables 4 and 5). IrisPlex model provided similar accuracy of prediction for blue eye colour when comparing to logistic regression and neural networks models developed in this study (IrisPlex AUC = 0.888, neural networks = 0.889, logistic regression = 0.872), slightly higher value of AUC for brown eye colour (IrisPlex AUC = 0.935, neural networks AUC = 0.917, logistic regression AUC = 0.889) but lower value for green (intermediate) eye colour prediction (IrisPlex AUC = 0.556, neural networks AUC = 0.667, logistic regression AUC = 0.611) (Fig. 2).

**Table 4:** Success rate of eye colour prediction in females and males based on six IrisPlex SNPs (IrisPlex online tool)

Prediction success based on IrisPlex model						
Eye colour category	Sensitivity %			Specificity %		
	Females	Males	*χ* ^2^*p* value	Females	Males	*χ* ^2^*p* value
Blue	95.1 (274/288)	91.8 (224/244)	0.117	71.8 (222/309)	76.1 (134/176)	0.304
Intermediate	0.0 (0/85)	0.0 (0/42)	–	100.0 (512/512)	100.0 (378/378)	–
Brown	88.0 (197/224)	91.0 (122/134)	0.363	89.5 (334/373)	88.8 (254/286)	0.797

**Table 5:** Success rate of eye colour prediction in females and males based on rs12913832 (IrisPlex online tool)

Prediction success based on rs12913832						
Eye colour category	Sensitivity %			Specificity %		
	Females	Males	*χ* ^2^*p* value	Females	Males	*χ* ^2^*p* value
Blue	94.1 (271/288)	91.0 (222/244)	0.170	73.8 (228/309)	79.0 (139/176)	0.200
Intermediate	0.0 (0/85)	0.0 (0/42)	–	100.0 (512/512)	100.0 (378/378)	–
Brown	90.2 (202/224)	92.5 (124/134)	0.449	88.5 (330/373)	87.1 (249/286)	0.583

## Discussion

DNA-based prediction of human appearance is a very promising approach that can be useful for investigations of biological traces and human remains when other DNA analysis methods fail to identify a suspect. However, high complexity of genetic basis underlying human appearance traits makes this field very difficult. The genetic prediction of human eye colour is the most thoroughly studied and the most advanced. The first eye colour prediction attempts have been reported before genome-wide association studies (GWAS) identifying large number of pigmentation-associated loci [20, 21]. The discovery of rs12913832 in *HERC2* has been crucial [22–24]. This SNP is believed to be a key eye colour regulator influencing *OCA2* expression. So far, the IrisPlex system is the most validated tool for eye colour prediction [4, 5, 12, 13, 19, 25–28]. This method is based on six eye colour prediction SNPs [6, 18] and provides high level of prediction accuracy for blue and brown eye colour at ~90 % with much lower accuracy level obtained for intermediate irises.

The IrisPlex validation studies, which have involved various populations, provided unprecedented insight into the genetics of the six IrisPlex predictors revealing significant inter-population differences in prediction capacity of particular SNPs. The key role of rs12913832 in eye colour prediction is undisputable, and clearly this marker is included in all available eye colour prediction models [6, 8, 10, 29]. The role of rs1800407 in *OCA2* has also been emphasized by many research reports [20, 30–35]. The effect of rs12896399 on eye colour determination discovered in a GWA study from 2007 [36] has also been confirmed in several studies performed on northern and southern European populations [8–11, 18]. Significance of rs16891982 in *SLC45A2* seems to depend on a minor allele frequency in a study population as it is lower in northern Europe populations (MAF ~ 0.04, 1000 genomes) and higher in southern Europe (MAF ~ 0.18, 1000 genomes). Despite its low frequency in Poland (MAF = 0.027), its effect has been detected in the present study at the level of OR = 0.471 (or 2.12) for blue/non-blue eye colour but with lower significance than observed for rs12913832, rs1800407 and rs12896399. The role of the remaining two positions, namely rs1393350 and rs12203592 is the most puzzling as the MAF in each case is rather high (MAF = 0.24 for rs1393350 and 0.12 for rs12203592, 1000 genomes) while the pattern of association is ambiguous. These two SNPs were not associated with eye colour in our previous study involving smaller population sample [11]. In the present study, position rs1393350 was associated with blue/non-blue eye colour only in a multivariate association analysis, and the result was insignificant after correction for multiple comparisons. A weak association of *TYR* rs1393350 and the lack of association in case of *IRF4* rs12203592 with eye colour have been reported in other study exploring several European populations [8]. *IRF4* has not been included in an eye colour classification tree developed by Allwood et al. based on a population sample from New Zealand while the model involves rs1393350 in *TYR* [10]. Lack of association for these two SNPs has also been revealed in a recent study performed on a Portuguese population [14]. The examination of rs12203592 in *IRF4* in 12 European and Asian populations has revealed very weak effect of this SNP for iris colour prediction [12]. The minor allele frequency for rs12203592 in our population is lower (MAF = 0.087) than assessed for global European population (MAF = 0.12, 1000 genomes) which may be the reason for the lack of association detected. However, association at the level of OR ~ 2.0 was discovered for rs1800407 and rs16891982 characterized by even lower values of MAF (0.064 and 0.027, respectively). Study performed on a Spanish population sample has revealed marginally significant association of rs12203592 in *IRF4* with eye colour with the effect size of OR ~ 2.12 when adjusted for the remaining IrisPlex SNPs [9]. Our study sample was theoretically sufficient to detect association of *IRF4* assuming OR ≥1.78. Thus, OR ~ 1.17 may suggest the problem with statistical power to detect association between eye colour and rs12203592 in this study. Interestingly, *IRF4* shows ambiguous pattern of association with cutaneous cancers in various populations [37–41]. Pietroni et al. has reported three IrisPlex SNPs to be the only informative for eye colour prediction, and this set comprised rs12913832 in *HERC2*, rs1800407 in *OCA2* and rs16891982 in *SLC45A2* [13]. In our study, position rs12896399 in *SLC24A4* excluded in that study was ranked as the second most important factor for blue/non-blue eye colour and the third most important factor for blue/green/hazel/brown eye colour determination. The increase in AUC value illustrating accuracy of prediction was the most noticeable after the implementation of rs12913832 in *HERC2*, rs1800407 in *OCA2* and rs12896399 in *SLC24A4* into the developed prediction models and was significantly lower when the remaining three IrisPlex predictors were added. These results strongly support existence of inter-population differences in association patterns in Europe and that different polymorphisms may have different predictive power in different populations. Therefore, we conclude that the set of six SNP predictors selected in IrisPlex model is a good minimal set for eye colour prediction when dealing with a sample of unknown biogeographic ancestry. These inter-population differences are even more severe at the worldwide scale. Blue eye colour is mainly limited to European populations, but there is a strong evidence for convergent evolution of skin pigmentation in Europeans and Asians and significant role of different DNA variants within *HERC2-OCA2* region responsible for skin lightening in Europe and Asia [42–44]. Interestingly, inter-population differences in the prevalence of various *OCA2* alleles were recently shown in the region of East Asia, and the data suggested that the studied polymorphisms might have been selected independently in various populations of East Asia [45]. This observation further emphasizes possible population-specific role of various pigmentation related DNA variants.

Moreover, the studies have also disclosed a role of gender for eye colour prediction accuracy which can be population specific [9, 13]. Although, gender has been found not to improve prediction of eye colour when incorporated into the original IrisPlex model [15], and further studies of this subject involving various populations can be intriguing. There are few studies suggesting that males tend to have lighter eye colour comparing to females. The prevalence of blue eye colour in a Spanish population is quite low, and recently significantly lower proportion of blue-eyed females (8.5 %) has been reported comparing to blue-eyed males (14.7 %). In turn, Spanish brown-eyed females have been found to be more common (78.5 %) comparing to Spanish brown-eyed males (71.4 %) [9]. Gender effect on quantitative eye colour variation (hue and saturation) has also been noted in the study performed on the Dutch Europeans [46]. The association of gender with quantitative eye colour variation has also been reported for the Italian study sample also suggesting that females tend to have darker eye colour than males. However, in the same study, no association of gender with eye colour variation has been observed in Danish and Swedish population samples [13]. Our data indicated significantly higher proportion of blue-eyed Polish males (58.1 %) comparing to blue-eyed Polish females (48.2 %). Consequently, Polish females were found more likely to have green eye colour (14.2 %) comparing to males (10.0 %), but there were no significant differences between genders for hazel and brown eye colours. In our previous study, higher proportion of Polish blue-eyed males (60.9 %) has been reported comparing to blue-eyed females (52.3 %), but the result has been insignificant which may be explained by significantly lower number of samples analysed (N = 388) [47]. In this large dataset, gender was found to be significantly associated with eye colour, but small size effect was reported with males having ~1.5 higher odds for blue eye colour comparing to females. Gender was revealed to explain 1.3 % of variation in blue/non-blue eye colour which is higher than reported in the Dutch population (0.1 %) [15] but lower than calculated for the Italian sample (4.9 %) [13]; however, in both cases, calculations have been performed for quantitative eye colour. In the multivariate logistic regression analysis covering six IrisPlex SNPs and gender, gender was ranked as third (when blue/non-blue eye colour has been considered) or fourth (when blue/green/hazel/brown eye colour has been considered) most important factor.

Identified association of gender with eye colour variation has raised a dispute on its possible impact on eye colour prediction performance. The authors of the study performed on the Spanish population have noted that among CC homozygotes for *HERC2* rs12913832, which is believed to be a strong predictor of blue eye colour, 79 % of males were indeed blue-eyed and only 54 % of females were blue-eyed suggesting that females tend to have darker eye colour than males when dealing with CC genotype in *HERC2* [9]. Similar result but with lower disproportion was obtained in the present study. Among Polish rs12913832 CC homozygotes, 85.7 % of males were found to have blue eye colour, and significantly lower proportion of blue eye colour was observed in females with 77 %. Contrary to the results obtained for the Spanish sample [9], the authors of IrisPlex model have not observed the differences in eye colour prediction success between males and females when analyzed much larger number of samples including >5300 Dutch Europeans and >3800 Europeans. They also have not observed the improvement of eye colour prediction when gender has been incorporated into the original IrisPlex model [15]. In the present study, higher sensitivity level of blue eye colour prediction was obtained in females comparing to males, and the opposite effect was observed in respect to the specificity level but the results were insignificant. Moreover, the incorporation of gender into the neural networks model developed in our study had ambiguous impact on the prediction efficiency with small AUC increase detected for green, hazel and brown eye colour but decrease observed for blue eye colour. These results confirm that the effect of gender on eye colour prediction is rather small in the studied population.

It is unclear how gender can affect the differences in human eye colour variation, and no genes on human gender chromosomes are known to be associated with pigmentation. Moreover, the effect of gender on eye colour variation seems to be complex as it appears to be population specific, with stronger effect noted in southern Europe populations. It is also worth noting that most of the reported European genome-wide association studies on pigmentation have been conducted on northern European populations [22, 36, 42, 48, 49]. Therefore, it seems that more extensive studies on the genetics of pigmentation exploring southern European populations are needed in order to clarify the contribution of gender into the eye colour variation in humans. Additional research is also needed to verify the impact of gender on eye colour prediction in various populations including admixed population samples.

Besides, verification of the list of predictors included into the eye colour prediction models also different mathematical approaches could be tested as they may give different results. So far, multinomial logistic regression [6, 18], Bayesian approach [8, 19] and classification trees [10, 29] have been used to develop eye-colour prediction models. In this study, neural networks have been explored and provided higher values of AUC comparing to logistic regression corroborating our recent observation reported for the prediction of hair morphology in humans that neural networks approach may be a good alternative for the traditional parametric methods [50]. Comparison of our neural networks model with the multinomial logistic regression IrisPlex model indicated slightly lower prediction accuracy of brown eye colour but noticeably higher accuracy of green eye colour prediction using neural networks approach. Since the discovery sample set used to build NN model was smaller (~1 000) than for the IrisPlex model (~10 000), further studies are necessary to explore this problem more thoroughly.

In conclusion, *HERC2*, *OCA2*, *SLC24A4* and *SLC45A2* were found to be significantly associated with eye colour in the studied Polish population. Gender was ranked as the third most important factor in blue/non-blue eye colour determination. Its effect size was found to be small with males having ~1.5 higher odds for blue eye colour comparing to females, and the observed impact on eye colour prediction was small. The obtained results provided further evidence that the genetics of eye colour is population specific and indicates that further studies on eye colour prediction involving various population samples, and more complex mathematical approaches will be intriguing.
